# LinkMind: Link Optimization in Swarming Mobile Sensor Networks

**DOI:** 10.3390/s110808180

**Published:** 2011-08-23

**Authors:** Trung Dung Ngo

**Affiliations:** 1 The More-Than-One Robotics lab, Department of Electronic Systems, Automation and Control Section, Aalborg University, Fredik Bajers Vej 7C, 9220 Aalborg, Denmark; 2 Faculty of Science, National University of Brunei, Jln Tungku, BE1410, Brunei

**Keywords:** optimization, max-flow min-cut, robot swarms, sensor network, line-of-sight communication

## Abstract

A swarming mobile sensor network is comprised of a swarm of wirelessly connected mobile robots equipped with various sensors. Such a network can be applied in an uncertain environment for services such as cooperative navigation and exploration, object identification and information gathering. One of the most advantageous properties of the swarming wireless sensor network is that mobile nodes can work cooperatively to organize an ad-hoc network and optimize the network link capacity to maximize the transmission of gathered data from a source to a target. This paper describes a new method of link optimization of swarming mobile sensor networks. The new method is based on combination of the artificial potential force guaranteeing connectivities of the mobile sensor nodes and the max-flow min-cut theorem of graph theory ensuring optimization of the network link capacity. The developed algorithm is demonstrated and evaluated in simulation.

## 1. Introduction

The swarming mobile sensor networks have received a lot of attention recently due to its challenges and potential applications. A network of mobile sensors can be utilized in hazardous environments for exploration and rescue mission, surveillance and reconnaissance, patrolling and monitoring, victim identification and data gathering [1–4]. Typically mobile wireless sensor networks have many advantages over wireless sensor networks because they are able to move for exploration, they can replace the fault nodes, they are able to adapt to the changes of environment rapidly, or they can carry different types of sensor for coverage and data collection. However, it is more difficult to deploy and manage a network of mobile nodes due to dynamics of network topological structure and mobility of mobile nodes, e.g., relocation of nodes transmitting messages through the network may prevent, interrupt, or break the link of information flow.

In real-world applications, there are two primary phases to apply a network of swarming mobile sensor nodes for data collection and propagation: *network deployment*, and *network preservation and optimization*. In the first phase, mobile sensor nodes are sent into the environment for a specific application. Basically, mobile nodes are deployed on the ground, over the sky, or under the water where they automatically communicate each other to form a large network. In many real-world environments, the nodes can talk to each other through line-of-sight communication (LoS) because most radio communication channels are disturbed by the environmental surrounding, e.g., metal shelves in the warehouse as in [5].

When a mobile node detects a source, it becomes a detector in the network and has responsibility to transfer the collected information to an operator. Hence, a network link of mobile routers between the detector and the operator must be established and their connectivities must be maintained for transporting messages in-between. Ideally, the link between the detector and operator, built through the ad-hoc network, is cohesively kept and its link capacity is maximized. This phase is named network preservation and optimization.

**Figure 1.**
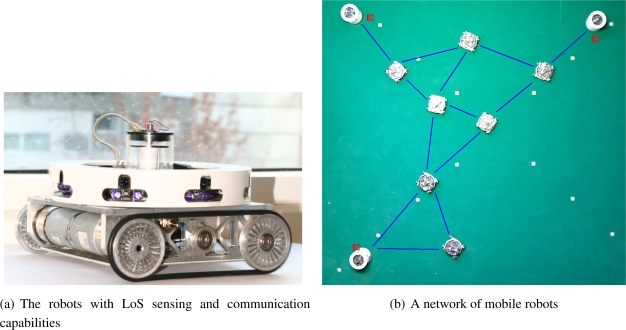
An example of the real robots and their network that needs to optimize the link capacity.

In the scope of this paper, we assume that the first phase has been done since the mobile sensor nodes have been already sent out by a deployment strategy. We only consider the second phase of how to preserve the link in-between detectors and operators and optimize the information flow. The former can be seen as the necessary condition guaranteeing a link through the network for information propagation while the latter is the sufficient condition ensuring the highest possible propagation of information in-between the detector and the operator as illustrated in Figure 1.

In the paper we employ combination of the artificial potential force (APF) [6] that creates a force field to keep mobile nodes connected and the graph theory based optimization method that improves the propagation of information from the source to the target. Note that one may say that artificial potential force alone may improve the network flow without the network link optimization. This claim might be true in some simple cases since the artificial potential force itself obtains local maxima or local minima that prevent the propagation of information through the network. Moreover, the artificial potential force only guarantees connectivities of the nodes that does not encompass the quality of network flow because the quality of network flow depends on utilization of the nodes when transmitting information through the ad-hoc network.

### 1.1. Artificial Potential Field

The artificial potential field was widely used in mobile robotics. It is originally coined out for single robot navigation [7] as the potential field represents interactions of the robot with obstacles in the environment. The potential field is decomposed into into attractive field and repulsive field. The attractive field aim at directing the robots to move towards a goal while the repulsive field enable them to move away from the obstacles. The sum of the attractive and repulsive fields is able to control the robot behaviors.

The artificial potential field has been extended into various ways to accommodate problems occurring in multi-robot systems and one of that is artificial potential forces. The potential force can be divided into two types, called environment-centered potential forces and robot-centered potential forces.

The environment-centered potential force is a global force that covers the whole navigation field in which obstacles by default are ascribed with repulsive forces and the goal is assigned with an attractive force. The robot is guided to follow the path found by the maximum of subtraction of those attractive and repulsive forces. Examples of the environment-centered potential force can be found in [7–9]. However, the drawback of the method is that it is not robust and flexible because the path is not changeable after being initialized by the global force field, therefore it may not be feasible to be applied for multi-robot systems whose dynamics must be frequently revised.

The robot-centered potential force represents the local influence of potential force to other robots and the environment [3,6,10,11]. The forces are usually based on local perception and/or inter-communication in which the attractive force pulls the robots close together while the repulsive force pushes the robots away from obstacles or other robots. The advantage of the robot-centered potential force is that the force is generated individually on each robotic agent so that it can be frequently updated. Because of this, the potential force field is used to develop controllers for robot swarms which require high robustness, flexibility and scalability.

A collection of artificial potential functions used to design the robot-centered potential forces in multiple robotic systems is categorized into: linear function, quadratic form, and exponential expression.

The light weight methodology [12] uses the Hooke’s law to create potential forces to maintain the robot formation. The social potential fields [10] is rather similar as it generates the inverse-power force laws between a pair of robots or a group of robots based on their social status corresponding to different forces between agents. In the trend of linear functions, the other potential force inspired by molecular-formed crystals was developed to enable formations of scalable multi-robot systems [13].

The Artificial Physics [6] is a typical representation of potential force based on quadratic function (although coefficients of the Artificial Physics can be chosen in another form). This artificial potential force was completely developed on individual agents and provides the systematic basic of self-organization, fault-tolerance and self-repair to the swarm. A similar approach was presented in [14] in which the potential force is described as a function of the distance from the robot to the goal and obstacles.

The work in [15] describes an approach to using exponential function to model attractive and repulsive forces. Our previous work [11] is also an example of the use of exponential function as potential force. It demonstrated that the developed forces are successful in keeping the robots in formation.

### 1.2. Graph Theory in Networked Systems

Graph theory is used to model networked systems, e.g., communication networks, and ad-hoc sensor networks. It is also applied to develop the model of networked robots in a large range of applications, e.g., coverage, synchronization of large-scale engineered systems, congestion control, and system performance. Usage of graph theory provides a variety of mathematical tools, methods and algorithms for abstracting and representing such systems.

Networked systems consist of a set of dynamical units that interact through information exchange for its coordinated operation and collaborative behaviours. Using graph theory, the coordinated or collaborated operation of such systems can be computed, controlled and even optimized w.r.t individual behaviours, collective decision of the group, and conditional states of inter-communication in-between units. In short, networked systems can be abstracted by a collection of vertices representing the units and a set of edges connecting two neighboring nodes representing connectivities and data flows.

In particular, a mobile ad-hoc sensor network is a type of networked systems. This is preferably exemplified as a graph of vertices and edges. Therefore the problems of mobile sensor network is converted to a graph theoretic problem. For example, the work described in [4] addresses the coverage of the sensor network that surmounts the local maxima when adapting to the environment by using the gradient climbing. In [16–18], stable flocking of mobile agents in both fixed, dynamic or switching topology are described and proven by techniques of graph theory. Formation control of multi-agent systems in [19,20] is also expressed in terms of the Laplacian. Distributed coordination control of multi-agent systems in [21] used the graph Laplacian for finding a non-linear feedback control ensuring the connectedness of mobile agents. In [22], the consensus problem of networked agents with capabilities of switching topology and time delays is modeled by weighted graph.

## 2. Problem Formulation

In this section we will define the optimization problem, specify delimitation, issue performance metrics and provide case studies for experimentation and evaluation.

### 2.1. Problem Definition

A graph *G(V,E)* is given to describe a network of mobile sensor nodes, where *V* is a set of mobile nodes and *E* is the set of links between them. *V* is categorized in three sub-sets: the sources *S*, the sinks *T*, and the intermediates *R*, where *S* and *T* must be non-empty.

In a network, each edge *e*(*v_i_*, *v_j_*) ∈ *E* is assigned a nonnegative capacity *c*(*v_i_*, *v_j_*) ≥ 0. The network flow is a communication channel that starts from a source *s* ∈ *S*, flows through intermediates *r* ∈ *R*, and be absorbed by a sink *t* ∈ *T*. On each intermediate *r*, the inflow *f_i_*(*r*) is the total of flows going into the node and the outflow *f_o_*(*r*) is the total of flows going out the node. Hence, for any *r* ∈ *R*, we have *f_i_*(*r*) = *f_o_*(*r*), and for each flow between two nodes *v_i_* and *v_j_*, the flow *f*(*v_i_*, *v_j_*) must satisfy the condition: *f*(*v_i_*, *v_j_*) ≤ *c*(*v_i_*, *v_j_*).

The value of a flow *f*, denoted *val*(*f*), is the total flow leaving the source *s* to the sink *t*, *val*(*f*) = ∑*_e_*_∈_*_Out_*_(_*_s_*_)_ *f*(*s*). Consequently, a *maximum flow* is defined as a flow *f^max^* fulfilling *val*(*f*) ≤ *val*(*f^max^*), ∀ *f*.

A partition of the network *G* is formed by *V_s_* and *V_t_* where source *s* ∈ *V_s_* and sink *t* ∈ *V_t_*. A *cut* in the network is a set of all edges that connect a vertex in *V_s_* to a vertex in *V_t_*. The capacity of a cut is a sum of the capacity of edges in the cut, denoted *c*(*V_s_*, *V_t_*). Hereafter, we define a *minimum-cut c^min^*(*V_s_*, *V_t_*) of the network as a cut with the minimum capacity.

In an ad-hoc network, to ensure existence of a flow starting from a source to a sink, the connectivities between nodes must be preserved. This task is done by the artificial force field to keep the robots in desired distance. Once the network of mobile nodes has been well established and maintained, finding a path with maximum flow is required to transfer information from the source to the sink as fast as possible. In graph theory, the Max-Flow Min-Cut theorem, so-called *mincut* [23] states that, for a given ad-hoc network, the value of maximum flow is equal to the capacity of a minimum cut, *f^max^* = *c^min^*(*V_s_*, *V_t_*). Consequently, to find the maximum flow of a network we can search for min-cut, which is also recognized as the bottleneck, of the network.

In a mobile sensor network in which nodes communicate with others via wireless communication channels, the link capacity between two nodes is inversely proportional to their relative distance [24]. Therefore, the link capacity is increased if the nodes are as close as possible. That is, the maximum flow of the network is increased by controlling the robots moving to desired positions where the total capacity of the minimum cut of the network is improved.

The main objective of this paper is to keep mobile robots swarming to form a network *G*(*V*, *E*) and to find min-cut, *c^min^*(*V_s_*, *V_t_*), of the network between sources *s* ∈ *V_s_* and sinks *t* ∈ *V_t_* in order to improve the maximum flow by manoeuvring the robots to desired positions in the network of mobile sensor nodes. Additionally, the network should adapt to the number of nodes added to the network (see definition of adaptability in Section 2.3). We define such a problem as *link optimization of swarming mobile sensor network*.

### 2.2. Delimitation

We focus on the problem of optimization by issuing delimitations. Two related terms named necessity and effect are stated to clarify each delimitation according to the characteristics of the mobile robots.

**Delimitation 1**: Robots shall be able to identify their immediate neighbors, maintain an in-memory map of all known neighbors, and locate itself in the network.

- *Necessity*: For identification robots are able to exchange identification (ID) of their immediate neighbors and get IDs of other robots being in the network to update its in-memory map of their immediate neighbors. For localization the robots are able to move according to the network demand.
- *Effect*: The robots with the low level of control must not only determine relative distance and direction of the neighbors but also communicate with the neighbors to maintain its in-memory maps over time. The robots are able to move to desired positions.

**Delimitation 2**. All nodes in the network must always be virtually connected. In other words, any node can always be reached by the others.

- *Necessity*: A search algorithm is needed to find out the other nodes in the network. This can be done by sending request signals through the network.
- *Effect*: The robots are expected to move to desired positions to optimize the network flow. However, the movement may affect to the quality of the network connectivity. The developed algorithm must preserve the network connectivity while controlling the robots to move to the desired positions. This implies the robots does not lose the connectivity to the network.

**Delimitation 3**: The robots shall be able to measure the on-the-fly flow running through them according to their positioning in the network and exchange those measurements in-between the nearest neighbors.

- *Necessity*: In order for mobile robots to relocate cooperatively to optimize the link capacity, each robot in the swarm must be aware of the overall flow running through it according to the network topology.
- *Effect*: A mechanism measuring the flow pressure on each robot must be developed. In simulation, it is defined as a measure factor.

### 2.3. Performance Metrics

To evaluate the developed algorithm, we define the following metrics.

- **Improbability**: The maximum flow should be improved over time after the networking link between two nodes in the swarming mobile sensor network is established.
- **Adaptability**: Adding nodes to the network should gain the maximum flow. That is, the network flow is increased if more intermediate routers is added.
- **Convergence**: The robot swarm should converge to a steady state solution of the maximum flow between two nodes in a certain scenario. That also means no more computing power is needed to control the robots’ mobility when the network flow is maximized.

### 2.4. Experimental Scenarios

We have chosen a number of scenarios as case studies to examine the developed algorithm. Some scenarios are fairly simple to illustrate the concept of network flow optimization while the other are problematic with a pitfall of the network or a local minima, which require a solution to improve the network flow.

In a scenario the robots are specified into two types: base stations (BS) and mobile nodes. The robots become the base stations if they are either sources or sinks, and they can not move. In contrast, the robots are mobile nodes if they are intermediate routers and are able to move.

**Figure 2.**
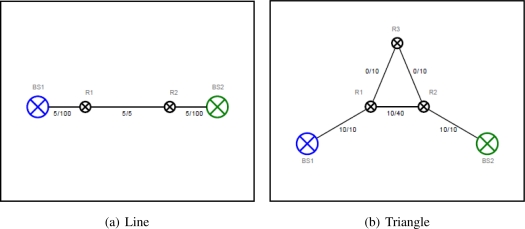
Case studies of connectivity priority.

In Figure 2(a) the two robots play a role as intermediate routers connecting the two base stations. Because the distance between the robots is shorter than the distance between the robots to the base stations on its side, the link between them is a bottleneck of the network. It is expected that the two robots are moving towards the middle to improve the link capacity.

In Figure 2(b) three robots connect to each other in a triangle topology and two of them are connected to the base stations. The communication capability between the two robots is greater than their communication bandwidth to the base stations. Thus the network link capacity is improved if the robots are moving towards the base stations. However the communication links of the two robots to the base stations and the other robot R3 have the same link capacity. Therefore, it is expected that the link between R1 and R2 is broken down and replaced by the intermediate router R3 so the overall network capacity between the two base stations is improved.

These scenarios are created to demonstrate the problem of connectivity priority for optimization.

**Figure 3.**
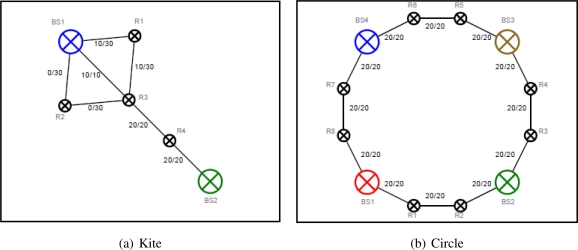
Case studies of local optimum.

In Figure 3(a) there is a redundant robot R2 participating but not contributing to the network on one end. There is a saturated link on the other end of the network. The link capacity between the base station BS1 and the robot R3 is lowest in the network, thus breaking the link and shifting the robot R3 towards BS2 will enhance the network capacity.

In Figure 3(b) the robots are placed in-between pairs of the base station to form a circle-like network. The scenario illustrates a class of local optimum, thus breaking the existing network and reassembling a new network topology are necessary to upgrade the overall network capacity. In order to improve the network capacity, the robots should temporarily break their link, move towards the center of the base stations, and form new connections.

These scenarios aims at demonstrating the problem of local optimum and the possibility of breaking links to improve the overall quality.

We have chosen two case studies where the Artificial Physics alone is impossible to improve the network quality because a robot is in the local minima.

**Figure 4.**
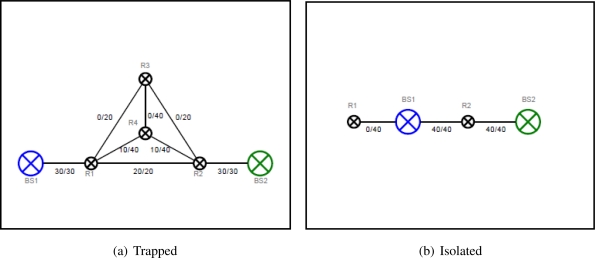
Case studies of local minima.

In Figure 4(a) the robot R4 is trapped in the center of a triangle of three other robots, which is a local minimum of the Artificial Physics. It is expected that the robot R4 can escape the position, and then moving down to the alignment of the two base stations to increase the bandwidth of the network.

Figure 4(b) shows the *Isolated* case in which a robot is hidden behind the base station and not connected to any other robots, thus it is not contributing to the network. It is impossible to use the Artificial Physics to relocate the robot to the other place where it contributes to the network because the base station is stationary and the robot is located in the ideal position corresponding to the base station. This robot position is also a typical case of local minimum when the Artificial Physics is applied. Hence, to improve the link capacity of the case study, an extended version of the Artificial Physics is required to overcome the local minimum.

**Figure 5.**
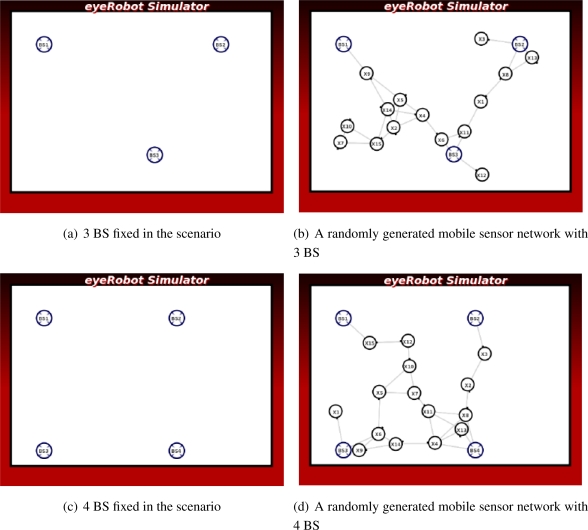
Two scenarios with randomly placed positions of the robots.

Instead of using a pair of source and sink, we wish to deal with the general case containing more than one source and one sink such that we must derive a super-source or a super-sink as seen later in 4.1. Two scenarios used to examine the case of super-sink, where there are three base stations in the first scenario and four base stations in the second one have been chosen as illustrated in Figure 5. A number of 15 robots are placed randomly in each scenario where all robots are virtually connected through a network. In each scenario, we execute 10000 simulation steps and measure the mean and variance of the maximum flow in each step.

As the number of the base stations and the robots are prescribed, the base stations are placed in fixed positions in the scenario, the average of maximum flow should converge to the same value for all 100 randomly generated setups. Thereby the mean of the maximum flow is an optimality while its variance implicates reliability of the scenario.

The differentiation between the first and the last simulation steps provides a comparative view of the improbability of the self-organized network.

The convergence criteria is reached if the network flow reaches a steady state over time. For each experiment of 100 randomly generated setups, the mean and variance are logged to assess the convergence.

To evaluate the adaptability of the network, we increase the number of robots from 9 to 21. the average maximum flow of the network in 100 randomly generated setups is measured and recorded. If the mean of the average maximum flow increases according to the number of networked robots, the adaptability criterion is considered successful.

## 3. The Relation of Artificial Potential Force and Network Link Capacity

Artificial Potential Force Field used widely to maintain the relative positioning between robots in a multiple robotic system can be categorized into two typical force fields: passive forces and active forces. Passive forces on each robot are independent constraints created by itself to restrict the robot mobility within its perceptional vicinity. The force field is artificially generated when the robots send out signals and measure the signal strength of the reflection, e.g., the distance to obstacles or neighboring robots by infrared, ultrasound, laser, or camera, for obstacle avoidance or relative distance maintenance. In contrast, active forces are externally influential factors which usually come from the other neighboring robots, e.g., communicative signals. The fact is that advantages of passive force field are disadvantage of active force field and vice versa: independent vs. dependent, non-neighboring awareness vs. neighboring awareness, non-distinguished vs. clearly distinguished between obstacles and robots.

Inspired from the robots depicted in Figure 1, we illustrate the concepts of passive and active forces in terms of infrared sensing and communication. Using infrared sensors, a robot can measure the relative positioning to other robots in two ways, named passive and active modes. In the passive mode, the robots send out signals and measure the strength of the reflected signals to perceive the surrounding, which are either obstacles or other robots. In the active mode, the robots capture the informative signal sent out from other robots to measure its strength to determine their relative positioning. Note that the active sensing is implicitly embedded in the communication between the robots, but their communication channel only exists if the robots are located within the communication range of the other robots.

One may employ both the passive and active modes to distinguish obstacles and other robots. Basically, the robots simultaneously send out the unique identification encoded signals and capture the incoming signals from itself if the signal are reflected from obstacles or from other robots. Based on the identification of captured signals, the robots can not only estimate the distance to surrounding but also distinguish the obstacle and other robots, which are extremely important in keeping the robots coherent and maintaining the network link through robot swarm.

The principle of light emitting sensors, e.g., infrared, is that the signal strength can be calculated by the ratio of inverse proportion of squared distance between the emitter and the receiver. This characteristic suggests that the potential force can be developed on the Artificial Physics [6]. The Artificial Physics is a kind of artificial potential forces in a quadratic form which was developed for connectivity preservation of robot swarm. The Artificial Physics consists of attractive forces pulling the robots closer when they are away each other and repulsive forces pushing the robots away from each other when they are close. There is a gap between attractive and repulsive forces, called neural force, in which the robots act freely only to maintain the networking linkage without considering their positioning. The equation of the artificial potential force on the robots in [6] can be seen in (1).

(1) $$\mathrm{APF}(v_{i},v_{j})=\frac{G*m(v_{i})*m(v_{j})}{r^{2}}$$

where the robots are denoted as *v_i_* and *v_j_*, their relative distance is denoted as *r*, and the gravitational constant that can be chosen at initialization is denoted as *G*.

**Figure 6.**
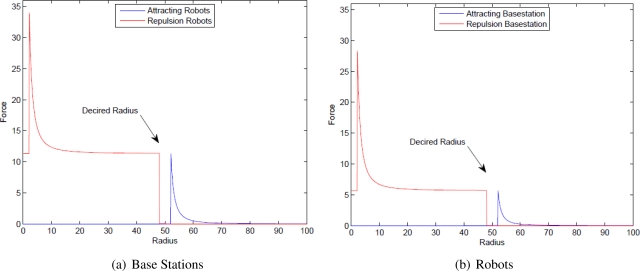
The Artificial Physics-based potential forces.

We developed two kinds of Artificial Physics corresponding to the functionality of the mobile robots. The Artificial Physics are automatically switched, depending on their role in the network, by using two different weighted factors. The robots acting as mobile routers are assigned the normal mass while the robots working as detectors when finding a source or as receivers when receiving information are assigned with a heavier mass, giving them higher priority to guarantee connectivity with the network as illustrated in Figure 6.

In a swarming network of mobile robots, one robot may connect with a number of other robots. The pseudo-code in Algorithm 1 shows how artificial forces of the neighboring robots are synthesized into a generalized force field.

The pseudo-code of switching modes between routing robots and base stations is presented in Algorithm 2.

**Algorithm 1:** Artificial Physics

1:	*combinedForce* := 0
2:	*combinedDirection* := 0
3:	**for***each neighbour***do**
4:	**if***neignbour*.*distance* > *desiredDistance***then**
5:	*force* = (*neigbour*.*mass* * *Gravity/neighbour*.*distance*^2^)
6:	*direction* = *neighbour*.*direction*
7:	**else if***neignbour*.*distance* < *desired Distance***then**
8:	*force* = *neigbour*.*mass* * *Gravity/neighbour.distance*^2^
9:	*direction* = *neighbour*.*direction*^−1^
10:	**else**
11:	*force* = 0
12:	*direction* = 0
13:	**end if**
14:	*combinedForce*+ = *force*
15:	*combinedDirection*+ = *direction*
16:	**end for**

**Algorithm 2:** Switching Mode

1:	**if***a source is detected or a target is assiged***then**
2:	*mass := big:mass*
3:	**else**
4:	*mass := normal:mass)*
5:	**end if**
6:	*call***algorithm** 1

In the ad-hoc network, two sensor nodes are capable of communicating with its immediate neighbors if they are mutually within the communication range with a distance, denoted *r*. If we use light emitting sensors, e.g., infrared board of the robots shown in Figure 1, *r* is also the limited range of the artificial potential force used to maintain the relative distance between the robots. In the Cartesian coordinate, the range *r* is calculated by (2), where coordinate of the robot *v_i_* is represented as $v_{i}^{x}$ and $v_{i}^{y}$.

(2) $$\mathrm{dist}(v_{i},v_{j})=\sqrt{{\left(v_{i}^{x}-v_{j}^{x}\right)}^{2}+{\left(v_{i}^{y}-v_{j}^{y}\right)}^{2}}$$

In simulation, the distance can be reformed for computation as in (3).

(3) $$\mathrm{dist}(v_{i},v_{j})=\sqrt{r_{v_{i}}^{2}+r_{v_{j}}^{2}-2r_{v_{i}}r_{v_{j}}\text{cos}(\theta_{v_{i}}-\theta_{v_{j}})}$$

where *θ_v_i__* is the heading of the robot *v_i_*.

Note that, in a directed graph *G*(*V*, *E*), only edges *E*(*v_i_*, *v_j_*) that are within the limited communication range *r* are taken into consideration of the link capacity optimization.

## 4. LinkMind Algorithm Development

When mobile sensor nodes are deployed in the environment, their network is represented as a graph of vertices connected by edges, *G*(*V*, *E*). If the nodes are connected, there exists a path from a vertex to the other vertex, thus the information sent out from a source vertex *s* is flowed through the other intermediate vertices *r_i_* : *i* ≥ 1 in the network and absorbed by a sink vertex *t*. When there exists a path between a source and a sink, the bottleneck of the path may be found using the Ford–Fulkersen method [25]. The method is well-known for finding the maximum flow of the network through a graph. An augmenting path between the source and the sink is iteratively ascertained and the flow along the path is incremented until no more links with spare capacity can be found.

In this paper, we use the Edmonds–Karp (EK) algorithm as shown in Algorithm 3, an extended version of the Ford–Fulkerson method. This algorithm uses the breadth-first search to find the shortest augmenting path between a source and a sink, which guarantees finding the maximum flow in a flow network in *O*(|*V*| · |*E*|^2^), instead the computational complexity, *O*(|*E*| · |f*|), using the depth-first search in the Ford–Fulkerson method depends on the maximum flow, f*, which is uncertain to be reached in a given graph as stated in [23].

**Algorithm 3:** Edmonds-Karp Algorithm

**Require:** (*s*, *t*)	
1:	**while** 1 **do**
2:	*path* := *BreadthFirstSearch*(*s*, *t*)
3:	**if***path* = 0 **then**
4:	*SourceSet* = *DepthFirstSearch*(*F*, *s*)
5:	*cut* := *GetEdgeBetween*(*SourceSet*, *InverseSourceSet*)
6:	(*maxflow*, *cut*)
7:	**end if**
8:	*flow* := *MinimumCapacityInPath*(*path*)
9:	*maxflow* := *maxflow* + *flow*
10:	*v* := *t*
11:	**while***v*! *= s***do**
12:	*u* := *path*[*v*]
13:	*F*[*u*, *v*] := *F*[*u*, *v*] + *flow*
14:	*F*[*v*, *u*] := *F*[*v*, *u*] − *flow*
15:	**end while**
16:	**end while**

### 4.1. The LinkMind Algorithm

The Edmond–Karp algorithm requires complete information of the link capacity over the network for calculation the minimum cut. We assume that robots through inter-communication can build in-memory map of positioning of all nodes and the link capacity of the network. Once the robots under control of the artificial potential forces are within the desired distance, the link capacity is an expression of the inverse Euclidean distance because the signal strength of light emitting sensors deteriorates over distance as calculated in 4.

(4) $$c(v_{i},v_{j})=f(dist{\left(v_{i},v_{j}\right)}^{-1})$$

However, in the scenario of multiple sources and sinks, called a multicommodity flow problem, the EK can not be directly applied. The EK maximum flow algorithm is executed if the scenario is modified with only one base station as a source and all the other base stations connected to an extra sink called the super-sink, where all extra edges are of infinity capacity. Consequently, the computational time of finding all min-cuts is increased with the number of base stations, *O*((|*V*| · |*E*|^2^)*^n^*) where *n* is number of sinks.

**Figure 7.**
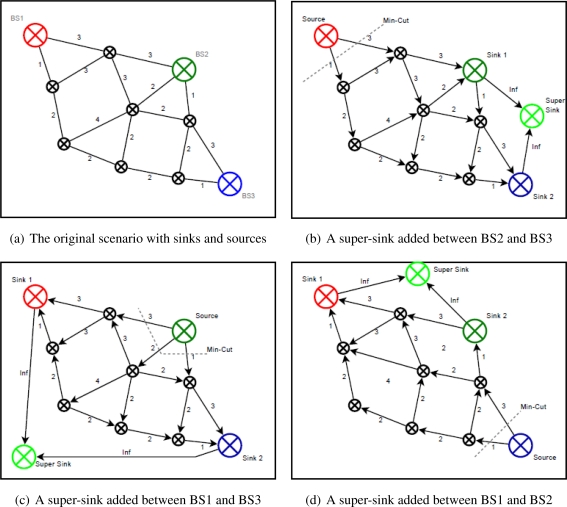
Super-sinks added in the scenario.

An example of super-sink can be seen in Figure 7. There are three base stations in the scenario corresponding to three independent graphs. The EK algorithm is executed only if a super-sink is connected to two base stations iteratively and the flow is run from the another base station.

Once the maximum-flow calculated by the Edmond–Karp algorithm has been found, the robots involved in the min-cut should relocate to improve the min-cut. Inspired by the Artificial Physics, a force vector is created to force the robots to move closer towards min-cuts, thereby the link capacity is improved.

On a robot *v_i_*, *MC*(*v_i_*) is a set of robots involved in the min-cut of *v_i_*. We let *MCF*(*v_i_*) be the min-cut force vector. We also define *V* (*v_i_*, *v_j_*) as the vector in Cartesian coordinates between two nodes involved in the min-cut of *v_i_*. Now the min-cut force vector *MCF*(*v_i_*) on the robot *v_i_* is a sum of forces of all robots in *MC*(*v_i_*), whose values are the inverse of their capacity as stated in (5).

(5) $$\mathrm{MCF}(v_{i})=\sum_{v_{j}\in MC(v_{i})}V(v_{i},v_{j})*c{\left(v_{i},v_{j}\right)}^{-1}$$

The algorithm of the min-cut force vector can be seen in Algorithm 4

**Algorithm 4:** Min-Cut Force Algorithm

1:	*forceV ector* = (0, 0)
2:	**for***cut* := *minCutEdges***do**
3:	*forceV ector = forceV ector + cut*.direction * (1/*cut*.*capacity*)
4:	**end for**
5:	**return***forceVector*

However, the min-cut forces are not able to maintain the connectivity of the robots due to uncertainty of information flow. We therefore still need the assistance of the Artificial Physics force to keep the robots coherent in the network. Now the force vector of the robot control is synthesized of two forces, the Artificial Physic based force stated in (1) and the min-cut based force stated in (5). The sum of these two forces are normalized with their coefficients in (6), depending on the characteristics of the communication mechanism.

(6) $$F(v_{i},v_{j})=n_{\mathrm{MC}}*\mathrm{MCF}(v_{i},v_{j})+n_{\mathrm{AP}}*\mathrm{APF}(v_{i},v_{j})$$

**Figure 8.**
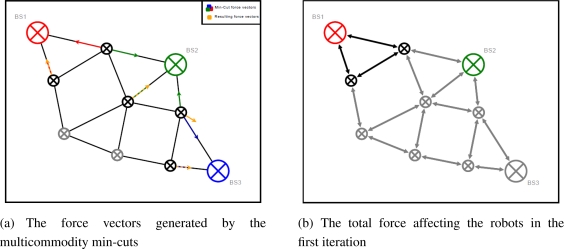
Force vectors from multicommodity min-cut problem.

Figure 8 illustrates an example of two robots which may lose connectivity with the network. However, when the Artificial Physics based forces are applied, these robots tend to follow the min-cut without knowing about it.

Once the general force in (6) is applied on every robot in the network, the robots will move towards the min-cut. To ensure the optimization process, the min-cut must be frequently updated according to the dynamical change of the network topology over time, which is illustrated in Algorithm 5

**Algorithm 5:** Update Min-Cut Algorithm

1:	**for***each BaseStation***do**
2:	*cut* = *EdmondsKarp*(*BaseStation*)
3:	**for***each robot containing edges of the cut***do**
4:	*addMinCut*(*cut*)
5:	**end for**
6:	**end for**
7:	**for***each robot***do**
8:	*get mincut*
9:	*move according to the force*
10:	**end for**

### 4.2. Experiments and Results

In this section, we examined the developed algorithm to identify and optimize the bottleneck in the representative scenarios.

**Figure 9.**
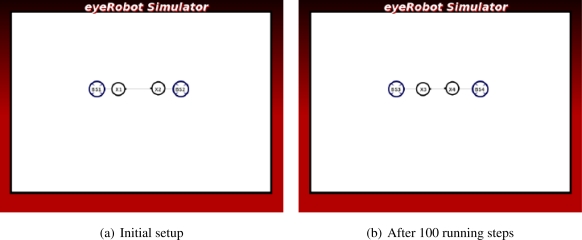
The experiment of Line scenario.

**Figure 10.**
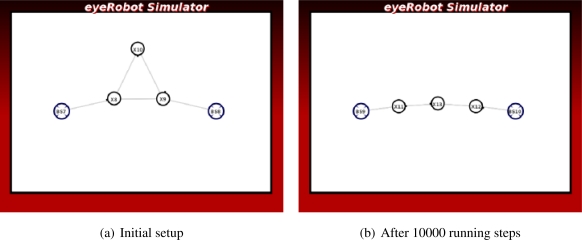
The experiment of Triangle scenario.

The experiments in Figures 9 and 10 show that the algorithm can improve the network flow between two base stations. In the *Line* scenario, two robots get close to each other to increase their link capacity. The robot, which is not directly contributing to the network, now joins the network after the link made with the other robots has been broken. It becomes an information carrier in the network of intermediate routers, thus the average maximum flow of the network is improved.

The force does not prioritize neither the base stations nor the robots, which is important to locate the robots equally between the base stations for the maximum flow.

**Figure 11.**
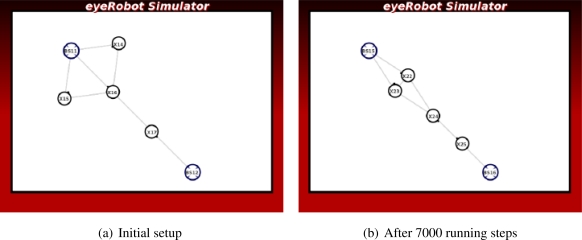
The experiment of the Kite scenario.

In the *Kite* scenario, the network flow is saturated with the initial setup in Figure 11(a). To improve the network traffic, breaking the link between the robots connected to the base station may be a good solution.

We have done the experiment with 7000 executions. The network at the final stage shows that the link between the two robots is broken up and the redundant robot moves towards the middle, and the average maximum flow is significantly improved as shown in Figure 11(b).

**Figure 12.**
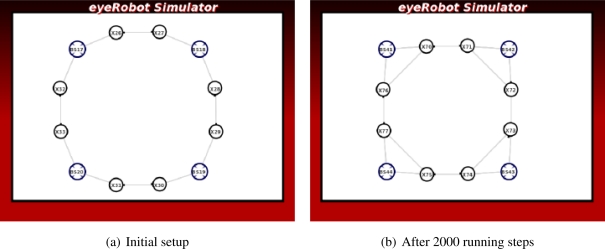
The experiment of the Circle scenario.

In the *Circle* scenario seen in Figure 12, the synthesized force can only make redundant links of two robots connecting the base stations and change the circle topology into the square-like topology. It can not break the links and move the robots to the center of scenario, thus the network flow is not further improved.

**Figure 13.**
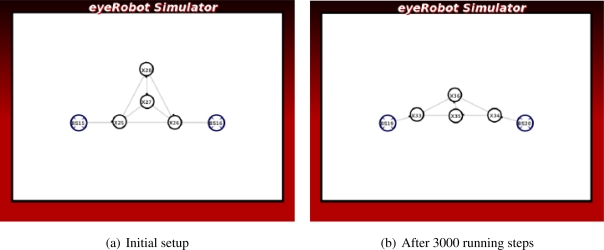
The experiment of the Trapped scenario.

The *Trapped* scenario as illustrated in Figure 13 is a special case study where the Artificial Physics can not solve the problem alone because the robot in the center of the triangle of the three other robots is in a local minima. In this case, two robots are redundant as they are not contributing to the network traffic. It is expected that the newly generated force will break the link between the two robots connected to the base stations and the other robots will move down to take a place at the broken link.

We have done the experiment in 3000 simulation steps and the achieved result is rather impressive. The link is broken and these two robots are directly involved in routing messages between two base stations. The overall network flow is significantly improved.

**Figure 14.**
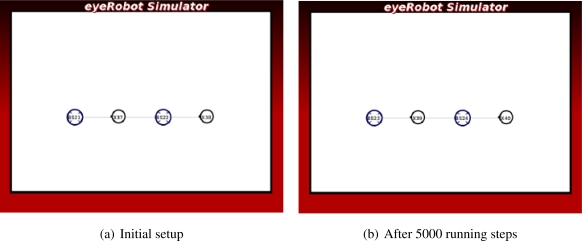
The experiment of the Isolated scenario.

We also examined the other special case, the Isolated scenario as shown in Figure 14, where the robot is in a optimal distance to the base station according to the Artificial Physics, but not a part of any network traffic inter-connecting base stations. This is called the local optimum of the Artificial Physics.

No minimum cut is detected on this robot and it is still located in the same position. As a result, the overall network flow is not improved.

**Figure 15.**
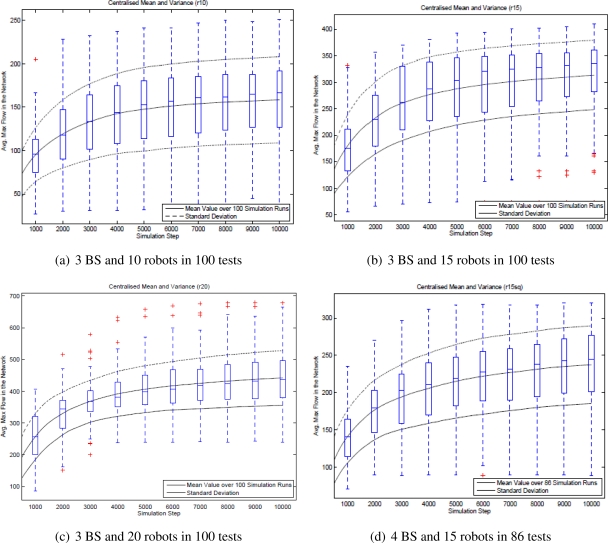
Average maximum flow of four setups.

To verify the performance metrics in terms of convergence and improbability, we have also examined the four setups in Figure 5. The results shown in Figure 15 confirms that the network flow is well improved over time in all setups. However, the network does not converge to a steady state in the four cases as the average maximum flow still increases over 10000 steps.

Finally, we examined the adaptability criterion by doing a large number of complicated experiments. The number of robots is increased from 9 to 21 and 100 setups with randomly placed robots are generated according to the chosen number of robots. The maximum flow with chosen number of the robots is averaged for comparison.

**Figure 16.**
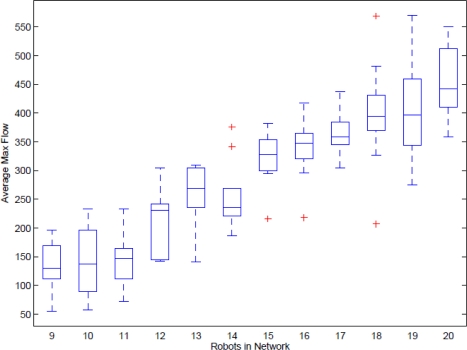
Average maximum flow with participation of 9 to 21 robots.

The result illustrated in Figure 16 shows that the average maximum flow is increased when adding more mobile routers in the network. This confirms that the developed algorithm is adaptable to the number of robots.

## 5. Discussion and Conclusions

Through several experiments we demonstrated that the LinkMind algorithm overcomes the local minima and local optimum problem of the Artificial Physics and improves the network flow in all the scenarios, except for the *Isolated* case. The bottleneck of the network can be found, and the network flow is optimized.

We believe that if a random walk is used in the case *Isolated*, the robot is able to escape from the local maxima, and then it moves to the position between the two base stations to improve the link capacity under the guidance of the LinkMind algorithm. However, this approach is not seriously considered as it is out of the scope of this paper.

Results presented in Figure 15 show that the average maximum flow is improved over time in all four tests. That is, the bottlenecks of the network link between the source and the sink are found and the robots under the guidance of the LinkMind algorithm relocate to improve the overall network flow. Therefore, the improbability criterion is successfully fulfilled with the developed algorithm. The variance of the network flow, however, seems to be rather large, meaning that the algorithm might not deal with the topological dynamics of network well. We may need to further consider properties of the algorithm to increase the reliability of network according to varying topology.

The achieved results shown in Figure 16 illustrated that the average maximum flow of the entire network is proportional to the number of robots contributing to the network flow. The adaptability criterion is successfully reached.

As seen in Figure 15, although the average maximum tend to converge to a steady states in all the four cases, none of those completely does. It would require excessive computational power of the robots to obtain knowledge of the entire network when more robots are added to the network, or more communication links are emerged through the network (meaning the number of base stations is increased). Hence, the convergence criteria is not fully satisfied with the developed algorithm.

The LinkMind algorithm should be recognized as a global optimization method since all robots must know about the positioning and information flow of the entire network through inter-communication. However, the requirement of available knowledge of the entire network is not feasible with a large-scale network of mobile nodes because the number of nodes contributing to the communication channel of a network flow might be varied due to dynamics of the environment. However, with a small or medium-scale network of mobile nodes, e.g., less than 30 nodes, this solution can be sufficiently satisfied since the robot swarm can be easier to maintain their connectivities, then communicate through the entire network for knowledge updating.

The paper has presented the development, experiments and evaluation of the LinkMind algorithm for optimization of the network flow capacity of a swarming mobile sensor network. The algorithm is based on the combination of artificial potential force and graph theory. Various experiment scenarios have been developed for experimentation and evaluation. Using the proposed performance metrics, we have proven that the algorithm is sufficiently powerful to find the minimum cuts of the mobile sensor network to improve the network flow capacity. This algorithm allows the robots to adapt to the network growth. Unfortunately, the convergence is not entirely fulfilled, causing the limitation of scalability of the network. Therefore, in the near future, we are considering a new solution about fully distributed algorithm, which not only preserves the highlights of the LinkMind algorithm but also provides superiorities to prevail over the existing drawbacks.
